# Delayed admission to the ICU is associated with increased in-hospital mortality in patients with community-acquired severe sepsis or shock

**DOI:** 10.1186/cc13431

**Published:** 2014-03-17

**Authors:** A Schnegelsberg, J Mackenhauer, M Pedersen, H Nibro, H Kirkegaard

**Affiliations:** 1 Aarhus University Hospital, Aarhus C, Denmark

## Introduction

The aim of this study is to determine whether ward transfers causing delayed ICU admission are associated with increased in-hospital mortality in patients with community-acquired severe sepsis or shock. Community-acquired infections are among the leading causes of ICU admission [1]. The 30-day mortality for adult emergency department (ED) patients with a delay in ICU admission of up to 24 hours has been found to be significantly higher compared with patients admitted directly to the ICU from the ED [2].

## Methods

A retrospective cohort study of patients admitted with community-acquired sepsis to a 12-bed, tertiary ICU at a university- affiliated teaching hospital, November 2008 to October 2010. Patients were divided into two groups based on their ICU admission pattern: direct transfer from the ED (direct group); and one or more ward transfers between the ED and ICU within 48 hours (delayed group). Our primary outcome measure was mortality.

## Results

We identified 277 patients admitted to the ICU within 48 hours from arrival in the ED. Of these, 186 were admitted directly from the ED, and 91 patients had more than one ward transfer between the ED and the ICU. In-hospital mortality in the delayed group was 32% compared with 21% in the direct group (*P *= 0.0477). Patients with delayed admission had significantly lower APACHE II scores: 21 (16; 26) and 24 (18.75; 31) respectively (*P *= 0.0016). The in-hospital LOS was similar in the two groups. For patients in the delayed group, we found a trend toward increased 30-day mortality (*P *= 0.0723), 90-day mortality (*P *= 0.0838) and higher Charlson Comorbidity Index (*P *= 0.0609).

## Conclusion

We found that patients admitted with community- acquired severe sepsis or shock are more likely to die in-hospital if they experience redundant ward transfers within 48 hours of admission. However, the delayed group was significantly lower risk stratified in the ICU based on their APACHE II score. Identification of severe sepsis and risk assessment in the ED is crucial for patient outcome.
